# Simulating an ultra-broadband concept for Exawatt-class lasers

**DOI:** 10.1038/s41598-020-80435-6

**Published:** 2021-01-08

**Authors:** Zhaoyang Li, Yoshiaki Kato, Junji Kawanaka

**Affiliations:** Institute of Laser Engineering, Osaka University, 2-6 Yamadaoka, Suita, Osaka 565-0871 Japan

**Keywords:** High-field lasers, Ultrafast lasers

## Abstract

The rapid development of the optical-cycle-level ultra-fast laser technologies may break through the bottleneck of the traditional ultra-intense laser [i.e., Petawatt (PW, 10^15^ W) laser currently] and enable the generation of even higher peak-power/intensity lasers. Herein, we simulate an ultra-broadband concept for the realization of an Exawatt-class (EW, 10^18^ W) high peak-power laser, where the wide-angle non-collinear optical parametric chirped-pulse amplification (WNOPCPA) is combined with the thin-plate post-compression. A frequency-chirped carrier-envelope-phase stable super-continuum laser is amplified to high-energy in WNOPCPA by pumping with two pump-beamlets and injected into the thin-plate post-compression to generate a sub-optical-cycle high-energy laser pulse. The numerical simulation shows this hybrid concept significantly enhances the gain bandwidth in the high-energy amplifier and the spectral broadening in the post-compression. By using this concept, a study of a prototype design of a 0.5 EW system is presented, and several key challenges are also examined.

## Introduction

Since the invention of the chirped-pulse amplification (CPA), where optical nonlinearity and damage in amplifiers are efficiently avoided, the peak-power of laser pulses has increased from Gigawatt (GW, 10^6^ W) to PW dramatically^1^. In the current PW lasers, the major laser amplification materials include Nd:glass, Ti:sapphire, and nonlinear optical crystal. The Nd:glass has a very large physical size (> 1 m) but a very narrow gain bandwidth (~ 20 nm FWHM), which has generated sub-picosecond, kilo-joule laser pulses with the current maximum peak-power of 2 PW^2^. The Ti:sapphire crystal has a limited physical size (< 200 mm) but a broad gain bandwidth (> 200 nm), which has generated 20–50 fs (limited by gain narrowing and gain saturation), < 400 J laser pulses with the current maximum peak-power of 10 PW^3,4^. In the optical parametric chirped-pulse amplification (OPCPA), for example the frequently used type-I Lithium triborate (LBO) crystal (pumped by 527 nm) also has a > 200 nm gain bandwidth but a limited physical size (< 150 mm). However, since the optical parametric amplification is free from the temporal-dependent gain saturation and gain narrowing, which can support < 20 fs high-energy ultra-short laser pulses with the current maximum peak-power of 5 PW^5^, and the construction of a 100 PW OPCPA laser facility has been started recently in Shanghai^6,7^.

**Figure 1:**
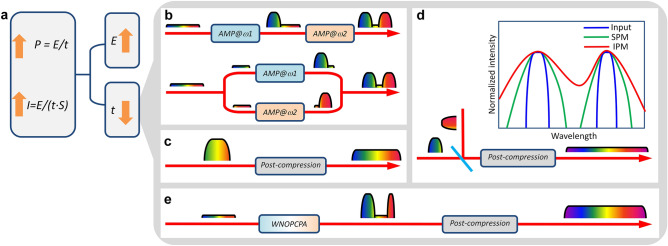
Approaches for peak-power/intensity scaling. (**a**) Two approaches of to increase pulse energy or to reduce pulse duration for peak-power/intensity scaling. *P*, peak-power; *I*, peak intensity; *E*, pulse energy; *t*, pulse duration; *S*, beam/focal-spot area. Ultra-fast optics methods to reduce pulse duration by broadening spectrum of (**b**) coherent pulse synthesis with sequential (upper) or parallel (lower) scheme, (**c**) post-compression in nonlinear medium [mainly by self-phase modulation (SPM)], (**d**) induced-phase modulation (IPM) with two different input spectra, and (**e**) concept in this paper of combination of WNOPCPA and post-compression. The inset in (**d**) schematically illustrates broader-band spectrum by IPM than that by SPM only.

As the next step to the EW-class laser, Fig. 1a shows two approaches that can be considered; one is to increase the pulse energy, and the other is to reduce the pulse duration. The former approach is strongly dependent on developing large-sized laser amplification materials (especially Ti:sapphire and nonlinear optical crystals) or increasing the beamline number (facility scale). This approach has to face with technical, engineering, and economic issues, for example parasitic lasing in a large-sized Ti:sapphire amplifier. Currently, the rapid development of the optical-cycle-level ultra-fast laser technology makes the latter approach more and more attractive, and may break through this bottleneck.

Coherent pulse synthesis, mainly demonstrated by F. Kärtner et al., enables the generation of an optical-cycle-level, carrier-envelope-phase (CEP)-controlled light pulse by combining different-colored pulses from separate sources/amplifiers^8^. Figure 1b schematically shows, by modulating phase, two light pulses from the sequential (upper) or parallel (lower) scheme with different gain spectra are combined into a single ultra-broadband (ultra-short) light pulse. Table 1 lists various experimental results^9–17^, where CEP-stable sub-optical-cycle (1.9 fs) light pulses have been produced.

**Table 1:** Selected results of coherent pulse synthesis, post-compression and induced phase-modulation.

Coherent pulse synthesis				
Laser source (scheme)	Spectrum	Duration	Peak-power	References
EDFA (parallel)	0.9–1.4 & 1.6–2 μm	4.3 fs (1-cycle)	100 kW	^9^
EDFA + Ti:sapphire (parallel)	0.7–1 & 1–1.4 μm	3.7 fs (1.1-cycle)	-	^10^
GF-HCF (parallel)	0.35–0.5 & 0.5–0.7 & 0.7–1.1 μm	2.1 fs (0.88-cycle)	0.15 TW	^11^
OPCPA (parallel)	0.75–1.05 & 1.9–2.5 μm	3.4 fs (0.8-cycle)	4.5 GW	^12^
OPA (parallel)	0.52–0.7 & 0.65–1 μm	3.8 fs (1.6-cycle)	-	^13^
OPA (parallel)	0.5–0.75 & 0.65–1 & 1.2–2.3 μm	1.9 fs (0.4-cycle)	-	^14^
TCP-OPA (sequential)	0.5–1 μm	4.6 fs (1.6-cycle)	0.2 GW	^15^
TCP-OPA (sequential)	0.6–0.7 & 0.7–1 μm	4.4 fs (1.6-cycle)	16 TW	^16^
TCP-OPA (sequential)	0.7–1.5 & 1.6–2.5 μm	3 fs (0.6-cycle)	8 GW	^17^

Post-compression				
Nonlinear medium	Spectrum	Duration	Peak-power	References
GF-HCF	0.55–1 μm	4.3 fs (1.7-cycle)	0.2 TW	^22^
Ionizing gas	0.5–0.9 μm	3.8 fs (1.5-cycle)	0.3 TW	^23^
GF-HCF	0.5–1 μm	4 fs (1.6-cycle)	0.7 TW	^24^
GF-HCF	0.3–1 μm	1.2 fs (0.5-cycle)	0.2 TW	^25^
GF-HCF	0.5–1 μm	3.4 fs (1.5-cycle)	1 TW	^26^
Fused silica	0.5–1.05 μm	3.7 fs (1.5-cycle)	25 GW	^27^
Fused silica	0.45–1 μm	2.5 fs (1.15-cycle)	20 GW	^28^
Fused silica	0.4–1 μm	2.6 fs (1.1-cycle)	90 GW	^29^

Induced phase-modulation				
Nonlinear medium	Spectrum	Duration	Peak-power	References
GF-HCF	0.35–1 μm	2.6 fs (1.3-cycle)	1.4 GW	^40^
GF-HCF	0.27–1 μm	1.5 fs (0.65-cycle)	0.3 TW	^41^

*EDFA* erbium-doped fiber amplifier, *GF-HCF* gas-filled hollow-core fibers, *TCP-OPA* two-color-pumped OPA.

Post-compression (or named as re-compression, nonlinear compression, super-continuum generation, and so on), demonstrated by M. Nisoli and F. Krausz et al., is a very important technology to generate optical-cycle-level light pulses^18–21^. Figure 1c schematically shows that an ultra-short pulse is generated by broadening the spectrum in a nonlinear device (e.g., gas-filled hollow-core fibers, crystal-photonics fibers, gas-filled cells, thin-plate bulk materials, etc.) and compensating the induced temporal chirp with chirped mirrors. Table 1 also gives several experimental results^22–29^, where CEP-stable sub-optical-cycle (1.2 fs) light pulses have been obtained^25^. Recently the concept of post-compression, previously demonstrated with low-energy ultra-fast lasers, has been extended to high-energy lasers by A. Zheltikov and G. Mourou et al. in order to generate few-cycle, PW and even EW laser pulses, where spectral broadening is achieved with very large and very thin solid-state materials^30,31^. This approach of post-compression, for example with large-aperture thin films or thin plates, in ultra-intense lasers are called as thin-film compression (TFC) or compression-after-compressor approach (CafCA), which has been successfully demonstrated and studied in several ultra-intense lasers^32–37^.

In the post-compression, the spectral broadening is dominated by self-phase modulation (SPM), which can be further increased by stimulated Raman scattering (SRS) and cross-phase modulation (XPM). Another important spectral broadening mechanism is induced-phase modulation (IPM), as illustrated in Fig. 1d, where the interaction between copropagating two (or more) different-colored light pulses in a nonlinear device can generate a much broader-band spectrum than that by SPM only (i.e., injecting different-colored pulses into the same nonlinear device, separately)^38,39^. Table 1 gives two experimental results of IPM in gas-filled hollow-core fibers^40,41^, where a CEP-stable sub-optical-cycle (1.5 fs) light pulse has been demonstrated. In addition, IPM recently is also demonstrated in thin plates, and around 5.5 times spectral broadening is achieved^42^.

Comparing the above three approaches, coherent pulse synthesis can combine amplified light pulses with different colors, which usually is not continuous in spectrum due to amplifiers; post-compression can generate a continuous ultra-broadband spectrum; and IPM can further enhance the spectral broadening in post-compression. Combining the three approaches together, Fig. 1e shows the concept in this paper: WNOPCPA firstly amplifies two different spectral components from an ultra-broadband seed to high-energy, and post-compression secondly broadens the high-energy spectrum to ultra-broadband. This method can support high-energy, spectral-continuous, ultra-broadband, and sub-optical-cycle laser pulses, eventually enabling peak-power/intensity scaling.

WNOPCPA is an improved multiple-beam pumped OPCPA^43,44^, where the pump is shaped into several beamlets and imaged onto the nonlinear crystal with different propagating directions. In this case, since several phase-matching conditions are satisfied in a single-stage amplifier, different spectral components of the signal are amplified simultaneously^45^. From the spectral point of view, WNOPCPA can be considered as a natural sequential-scheme coherent pulse synthesis, where different-colored pulses from the same ultra-broadband signal are amplified in the same amplifier, and consequently the phase control here becomes much convenient, i.e., only the spectral phase and CEP controls are required and the relative delay is satisfied naturally (see requirements for coherent pulse synthesis in Ref.^8^).

Here, in the WNOPCPA, the total number of the pump-beamlets is reduced to only two, and a time delay is introduced between them to completely eliminate the interference of the multiple pump-beamlets in the nonlinear crystal^45^. In the post-compression, the nonlinear spectral broadening is enhanced by IPM, resulting in a high-energy, ultra-broadband continuous spectrum suitable for generating a high-energy, sub-optical-cycle laser pulse. In the following sections, we present detailed numerical results of the proposed concept composed of the state-of-the-art optical components, including amplification in the WNOPCPA, nonlinear propagation in a fused silica thin wafer, and dispersion compensation by the chirped mirrors. We also discuss the nonlinear effect in the chirped mirror and the intensity modulation in the post-compression. From these analyses, it is shown that a laser pulse of 589 PW peak-power and ~ 1.65 fs pulse duration with total energy of 971 J is generated with a square beam of 980 mm × 980 mm aperture. Several issues to achieve over 1 EW by extending the proposed concept are also examined.

## Results

### Conceptual design

**Figure 2:**
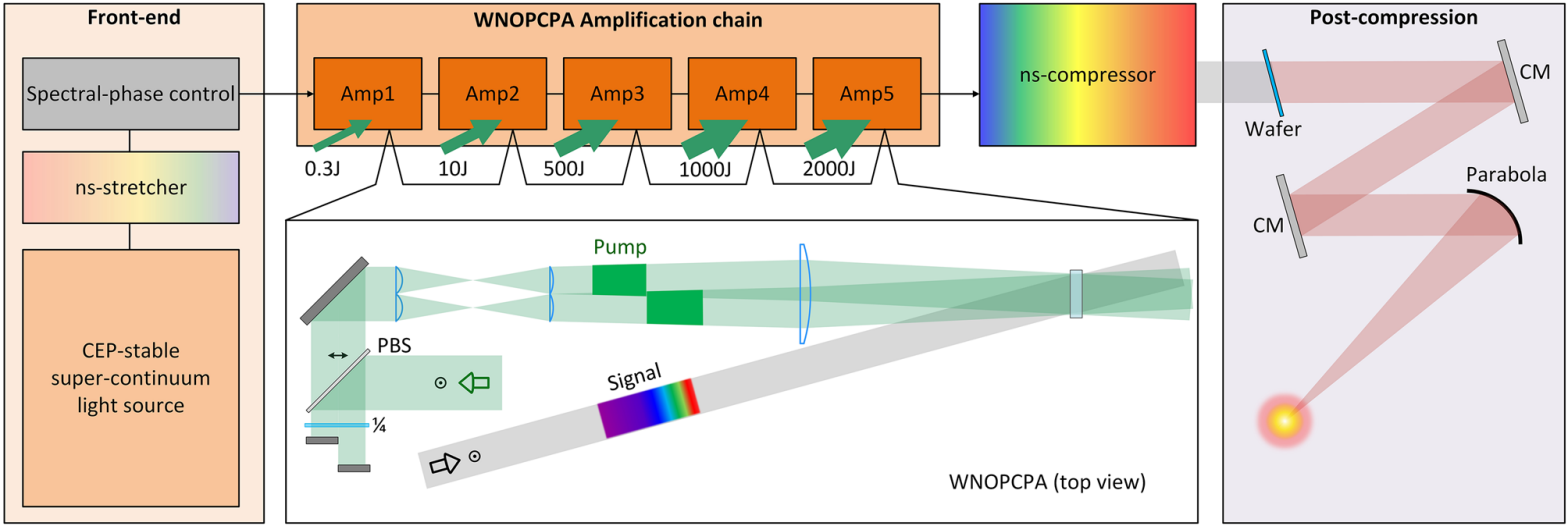
Schematic of the hybrid WNOPCPA and thin-plate post-compression concept. The front-end consists of a carrier-envelope-phase (CEP)-stable super-continuum light source, a nanosecond (ns)-stretcher and a spectral-phase control device. The amplification chain consists of five-stage of WNOPCPA for ultra-broadband amplification. The nanosecond (ns)-compressor compresses amplified chirped-pulses. The post-compression consists of a fused silica wafer for nonlinear spectral broadening, chirped mirrors (CM) for pulse compression, and a parabola for beam focusing. PBS, polarization beam splitter; and ¼, quarter wave plate. The inset shows the top view of a WNOPCPA amplifier.

Figure 2 shows the overall layout of the conceptual design of a 0.5 EW laser based on the hybrid system of WNOPCPA and post-compression. This system consists of the four parts; a front-end, an amplification chain, a nano-second compressor and a post-compressor. The front-end mainly includes a CEP-stable super-continuum light source, and almost all of the previous setups introduced in Table 1 can meet the requirement. The super-continuum pulse is stretched to a nano-second width with a grating stretcher, and sent to a spectral phase controlling device, e.g. an active controlling element of acousto-optic programmable dispersive filter (AOPDF)^46–48^ or a passive controlling element of grism pair^49^ or both, to accurately adjust the spectral phase of the whole laser system to generate a Fourier transform-limited (FTL) pulse after the nano-second compressor. A temporal contrast improvement device, e.g. a cross-polarized wave (XPW) device, could be also installed for generating an ultra-clean seed pulse^50,51^. This broad-band nanosecond pulse is sent to the WNOPCPA amplification chain as the signal beam for optical parametric amplification.

The WNOPCPA amplification chain includes five-stage amplifiers of Amp 1-Amp 5. As shown in the inset of Fig. 2 for one of the amplifiers (top view), the pump beam is divided into two beamlets and imaged onto the nonlinear crystal by a pair of cylindrical lens-arrays and a cylindrical Fourier lens. Because WNOPCPA has two pumping directions in this configuration, two phase-matching conditions are satisfied simultaneously, broadening the gain spectrum of the signal. A time delay is introduced between the two pump-beamlets to completely avoid the pump interference, which may otherwise modulate the signal beam and even damage the nonlinear crystal. Since the two-beamlet pump is required only in the phase-matching plane, a cylindrical (not spherical) lens-array pair and a cylindrical (not spherical) Fourier lens are utilized. For the image relay in the orthogonal propagation plane, another cylindrical lens pair can also be inserted into the setup, like the slit spatial filter developed for high-energy laser systems^52^. After five-stage WNOPCPA, the duration of the amplified high-energy ultra-broadband nano-second chirped-pulse is restored by a nano-second compressor formed by four large-aperture gold or hybrid gold-dielectric reflection gratings. The residual high-order dispersion is pre-compensated by the spectral phase controlling device at the front-end. The output of the near-FTL high-energy pulse is then delivered to the post-compression, where the spectrum is further broadened by the nonlinear propagation in a large-aperture thin glass wafer, and the generated chirped pulse is compressed by the large-aperture chirped mirrors. The compressed EW-class laser beam is sent to an experimental platform for exploration of ultra-high field, ultra-fast sciences.

### Broadband amplification with WNOPCPA

The five-wave (one signal, two pumps and two idlers) optical parametric coupling is simulated using the following parameters. In the spectral domain, the pump wavelength is 527 nm and the spectrum of the signal at least covers the range of 670–1130 nm. In the temporal domain, the pulse duration of each pump-beamlet is 1 ns with a 1 ns time delay between them resulting in the total duration of 2 ns for the two-beamlet pump, and accordingly the pulse duration of the chirped signal (670–1130 nm) is 2 ns. In the spatial domain, the signal and pump beams in Amp 1-Amp 5 have flattop square apertures with a side length of 5, 20, 130, 130 and 130 mm, respectively. The total energies of the pumps for Amp 1-Amp 5 are 0.3, 10, 500, 1000 and 2000 J, respectively, which in each amplifier are equally distributed to two pump-beamlets. In Amp 1-Amp 5, type-I LBO crystals are used, because the LBO crystal can be grown in larger-sizes and has higher damage-threshold compared with the BBO crystal^53^. In the type-I LBO (biaxial) crystal, the *x–y* plane is chosen as the phase-matching plane (*θ* = 90°), and the propagation angles *ϕ* (angles from the optical *x*-axis) of the two pump and one signal beams are 13.12°, 12.90° and 12.00° respectively. The optimization of the phase-matching is given in Supplementary material. Here, we choose the phase-matching direction with a smaller signal propagation angle (compared with those of two pumps), because the angle-gap between two pump-beamlets is enlarged^54,55^. These parameters together with other parameters are summarized in Table 2.

**Table 2:** Parameters in each amplifier from Amp 1 to Amp 5.

	Amp 1	Amp 2	Amp 3	Amp 4	Amp 5
Crystal type	Type I LBO				
Crystal aperture (mm^2^)	5 × 5	20 × 20	130 × 130	130 × 130	130 × 130
Crystal length (mm)	22.4	12.5	12	4.2	2.6
Pump spectrum (nm)	527				
Pump beamlet 1 angle (°)	*θ* = 90, *ϕ* = 13.12^a^				
Pump beamlet 2 angle (°)	*θ* = 90, *ϕ* = 12.90^a^				
Pump beamlet duration (ns)	1				
Pump duration (ns)	2				
Pump total energy (J)	0.3	10	500	1000	2000
Pump intensity (GW/cm^2^)	0.6	1.25	1.48	2.96	5.92
Signal spectrum range (nm)	670–1130				
Signal angle (°)	*θ* = 90, *ϕ* = 12.00^a^				
Signal duration (ns)	2				
Input signal energy (J)	0.0001	0.09	3.52	182	573
Output signal energy (J)	0.09	3.52	182	573	1,387

^a^For the phase-matching optimization, see Supplementary material.

**Figure 3:**
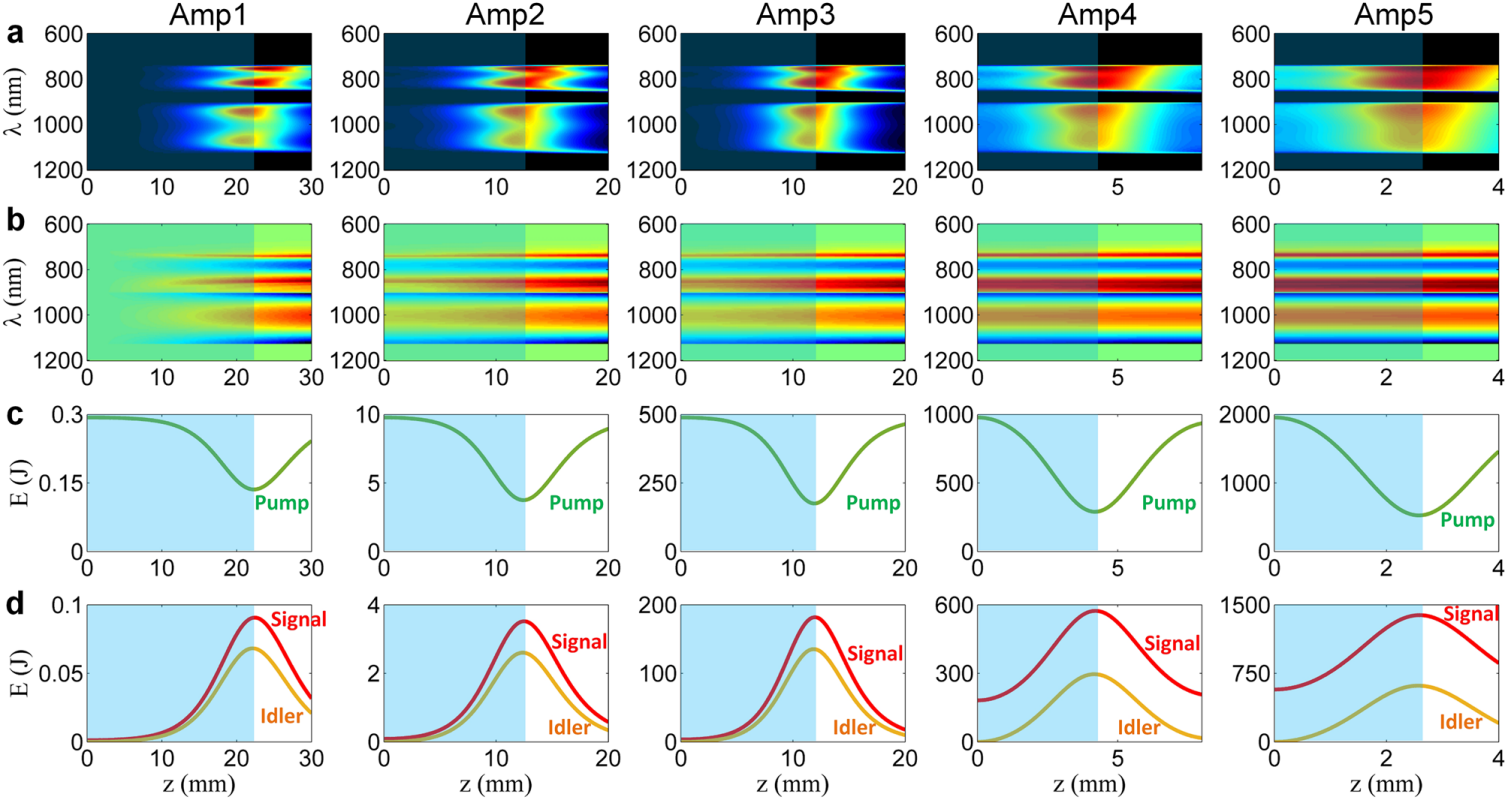
Evolution of the WNOPCPA amplification chain. Evolutions of (**a**) spectrum and (**b**) nonlinear spectral phase of signal, and energies of (**c**) pump and (**d**) signal and idler in nonlinear crystals along propagation *z* from Amp 1 to Amp 5. Blue areas show the optimized crystal lengths.

Figure 3 shows the simulation results of the evolutions of the spectral intensity and the nonlinear spectral phase of the signal beam, and the energies of the pump, signal and idler beams along propagation in the nonlinear crystals. Figure 3a shows the signal spectrum is composed of two separated components due to two discrete pumping directions, and Fig. 3b shows the corresponding nonlinear spectral phase (or called optical parametric phase). The linear phases due to dispersion in the stretcher and the crystal material have been removed in Fig. 3b, in order to directly observe the evolution of the nonlinear spectral phase of the signal. As shown in Fig. 3d, the energy of the signal beam becomes maximum of 0.09, 3.52, 182, 573 and 1,387 J in Amp 1-Amp 5 at the crystal length of *z* = 22.4, 12.5, 12, 4.2 and 2.6 mm, respectively. The highest efficiency of parametric amplification is obtained by optimizing the crystal thicknesses at these values. The energy of the signal beam at the output of Amp 5 reaches 1,387 J, which is 39.5% of the total pump energy of 3,510 J. The signal beam is expanded from 130 mm × 130 mm to 980 mm × 980 mm before the nano-second compressor to reduce the energy fluence to ~ 0.15 J/cm^2^ (on the beam cross-section) at the first grating and ~ 0.1 J/cm^2^ (on the beam cross-section) at the fourth grating, in order to avoid the possible damage of the fourth compressor gratings which have the measured damage threshold of around 0.15–0.8 J/cm^2^ (on the grating surface) for femtosecond pulses^56^.

**Figure 4:**
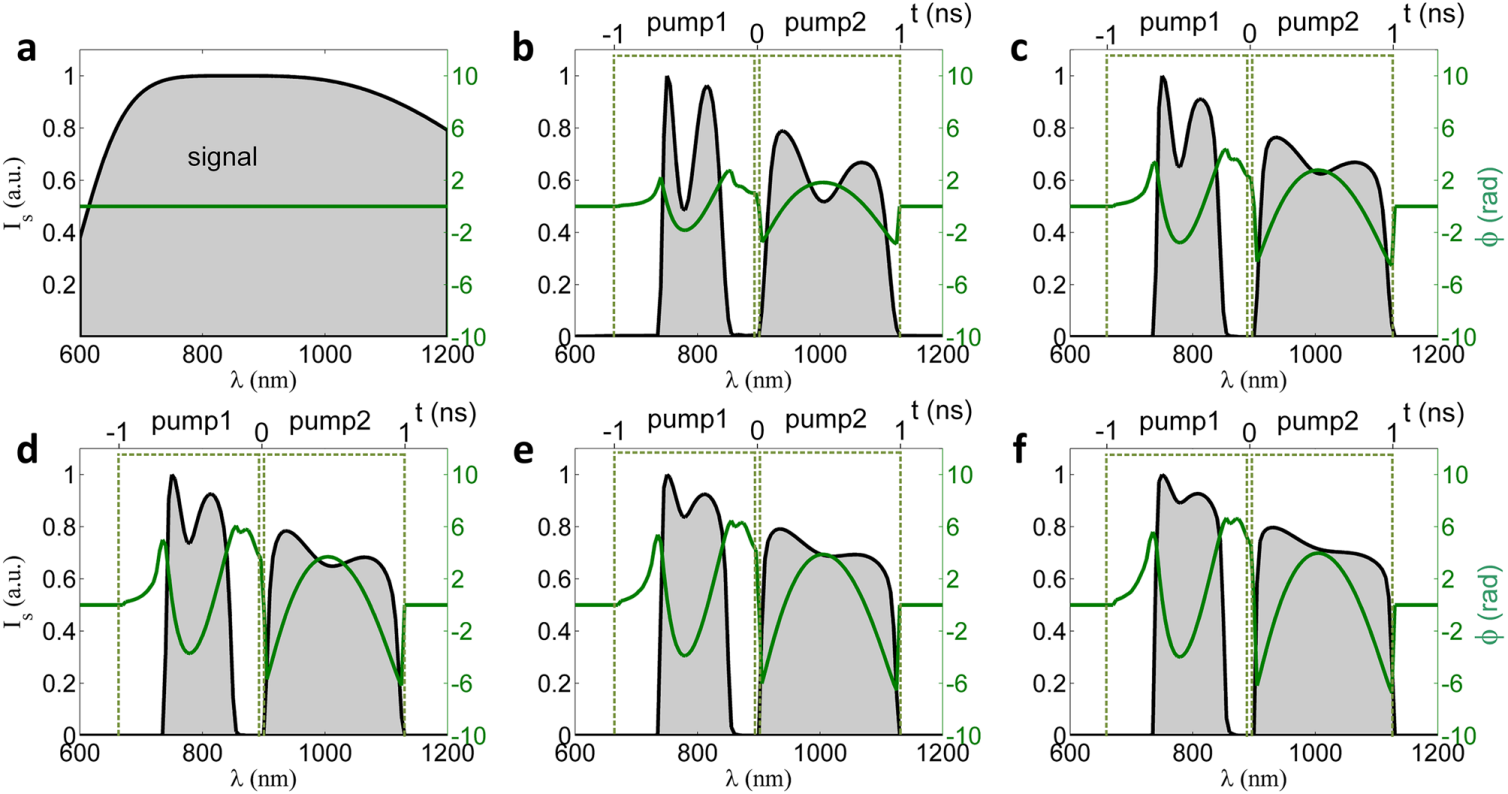
Spectrum and nonlinear spectral phase at each stage. Spectrum (black solid line) and nonlinear spectral phase (green solid line) of signal at (**a**) input and (**b**–**f**) outputs of Amp 1–5, respectively. Chirped signal pulses (black solid line) and two pump pulses (green dash line) in time are illustrated by upper labels. Pump 1 and pump 2 are two beamlets of the pump.

Figure 4 shows the spectra and the nonlinear spectral phases of the signal beam at the input to Amp 1 (i.e., output of the front-end) and at the outputs of Amp 1-Amp 5. The amplified signal has two separated spectral components of 730–860 nm and 900–1130 nm, corresponding to the two different pumping directions in WNOPCPA. Although the spectrum of the amplified signal is not continuous, these separated components are still coherent since they are amplified from the same coherent seed. The nonlinear spectral phase increases gradually from Amp 1 to Amp 5 and becomes approximately 4π rad (in peak-to-valley) at the output, and this spectral phase distortion is still within the control range of the available device for spectral phase control^46–49^. In the pulse compression, most of the temporal dispersion is removed by the nano-second grating compressor, and the residual high-order dispersion is pre-compensated by the spectral phase controlling device at the front-end. Referring to the available meter-sized gold or hybrid gold-dielectric reflection gratings, the throughput efficiency of the nano-second grating compressor is chosen as 70%. A FTL pulsed beam with a pulse energy of 971 J, a pulse duration (FWHM) of ~ 8.0 fs, a beam aperture of 980 mm × 980 mm, and a broadband spectrum as shown in Fig. 4f is injected to the post-compression.

The upper label of Fig. 4 also shows the pulse overlap between the pump (green dash line) and the signal (black solid line) in time. The pump 2 and the corresponding signal spectral component of 900–1130 nm perfectly overlaps in time. While due to a narrow gain bandwidth, the temporal overlap between the pump 1 and its corresponding signal spectral component of 730–860 nm is not high, which wastes part pump energy and accordingly reduces the conversion efficiency of the WNOPCPA. In order to compare this (low-energy) WNOPCPA with the (high-energy) OPCPA, in Supplementary material we have optimized type-I LBO-based and single beam pumped OPCPA with the broadest gain spectrum. Supplementary material also gives the re-simulation of a five-stage OPCPA amplification chain with the perfect pump-signal temporal overlap and the same pump energy of 0.3, 10, 500, 1000 and 2000 J in each amplifier. In this case, the amplified energy reaches 1940 J with the FTL pulse energy of 1358 J after the nano-second grating compressor (70% efficiency) which is higher than the amplified energy of 971 J obtained with WNOPCPA. These two different configurations of WNOPCPA (971 J) and OPCPA (1,358 J) are compared in the following post-compression.

### Spectral broadening with thin-plate post-compression

High-quality, large-aperture substrate wafers are available due to mass production in industry. Their thickness generally varies from 0.2 to 10 mm with the aperture sizes from 10 mm to 1 m and the surface roughness of less than 1 nm. The materials include fused silica, quartz, sapphire, and other glasses. Among these materials, a meter-sized fused silica wafer is very suitable for the post-compression of an ultra-intense laser due to its high damage-threshold, moderate nonlinear refractive index and dispersion coefficients.

The evolution of the high intensity FTL pulse in a fused silica thin wafer is analyzed for two inputs from 971 J output of WNOPCPA and 1358 J output of OPCPA, respectively. For comparison, we assume that the 971 J input have a square aperture of 980 mm × 980 mm and the 1,358 J input has a linearly increased square aperture of 1160 mm × 1160 mm (for the same energy fluence), all with flat-top beams but different spectra. The input intensity at the fused silica thin wafer cannot be too high (lead to conical emission and even dielectric breakdown) or too low (weaken spectral broadening), and here it is kept at around 10 TW/cm^2^, because at this intensity the multiphoton-induced plasma density in fused silica is safely below the documented avalanche plasma density^57^. The spectrum of WNOPCPA is shown in Fig. 4f, which possesses two spectral components of 730–860 mm and 900–1130 nm, while that of OPCPA has only one broadband spectral component of 765–1020 mm due to a single pump beam (see Supplementary material). The simulations are made with the following parameters: the center wavelength *λ*_0_ = 950 nm, the refractive index *n* = 1.4511, the GVD *β*_2_ = 24.759 fs^2^/mm, the TOD *β*_3_ = 34.840 fs^3^/mm, the nonlinear refractive index *n*_2_ = 3.67 × 10^–16^ cm^2^/W, and the Raman response time *T*_*R*_ = 4.2 fs^58^. Because the fused silica wafer is very thin (less than 1 mm), the attenuation *α* is chosen as 0. We use a constant Raman response time *T*_*R*_ of 4.2 fs for linear approximation in this paper, since the sensitivity of the spectral profile to slight variation of the Raman response time *T*_*R*_ is very low^31,58^.

**Figure 5:**
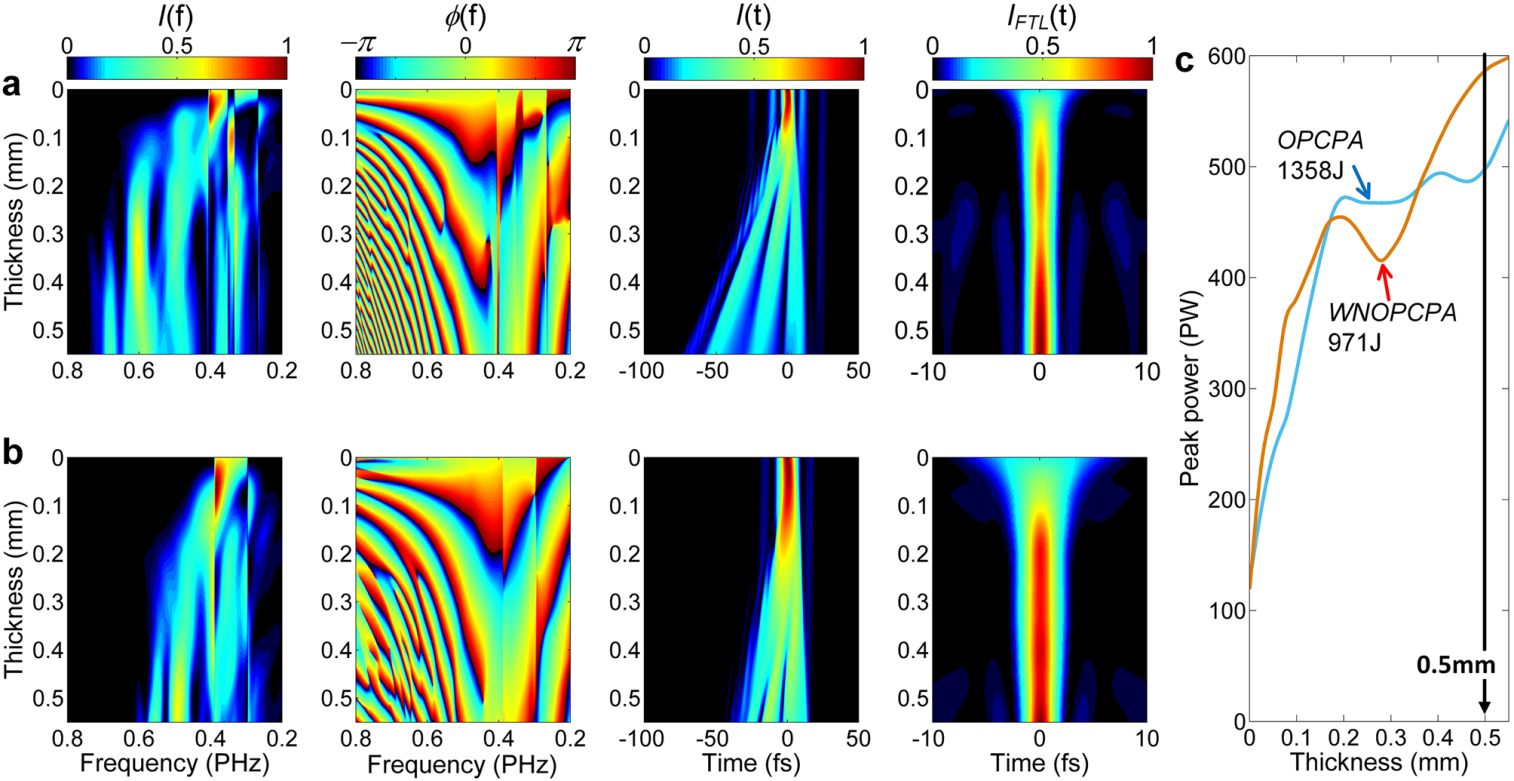
Nonlinear spectral broadening, pulse compression, and comparison. Evolution of spectrum *I*(*f*), spectral phase *ϕ*(*f*), pulse *I*(*t*), and the FTL pulse *I*_*FTL*_(*t*) during propagation in fused silica wafer for input pulses from (**a**) WNOPCPA and (**b**) OPCPA, respectively. (**c**) shows peak-power of the FTL pulse as a function of fused silica thickness for OPCPA (1,358 J) (blue) and WNOPCPA (971 J) (orange). FTL, Fourier-transform-limit.

Figure 5a,b show the evolutions of the spectrum *I*(*f*), the spectral phase *ϕ*(*f*), the pulse *I*(*t*) and the calculated FTL pulse *I*_*FTL*_(*t*) [calculated from the spectrum *I*(*f*)] during propagation in a thin fused silica wafer, for the FTL input pulses from WNOPCPA and OPCPA, respectively. Comparison of Fig. 5a,b shows that the spectral broadening is enhanced (due to the separated two spectral components of the input pulse), the pulse is more stretched (due to a broader spectrum) and the FTL pulse width is shorter (also due to a broader spectrum) for WNOPCPA, whereas the spectral phase evolutions for two cases are not significantly different. Figure 5(c) illustrates the dependence of the peak-power of the FTL pulse *I*_*FTL*_(*t*) on the thickness of the fused silica, which is expected after the perfect dispersion compensation. Although the peak-powers of the input pulses from WNOPCPA (971 J) and OPCPA (1358 J) are close to each other, the difference among the expected FTL peak-powers increases sharply with the thickness of the fused silica, especially after ~ 0.35 mm.

**Figure 6:**
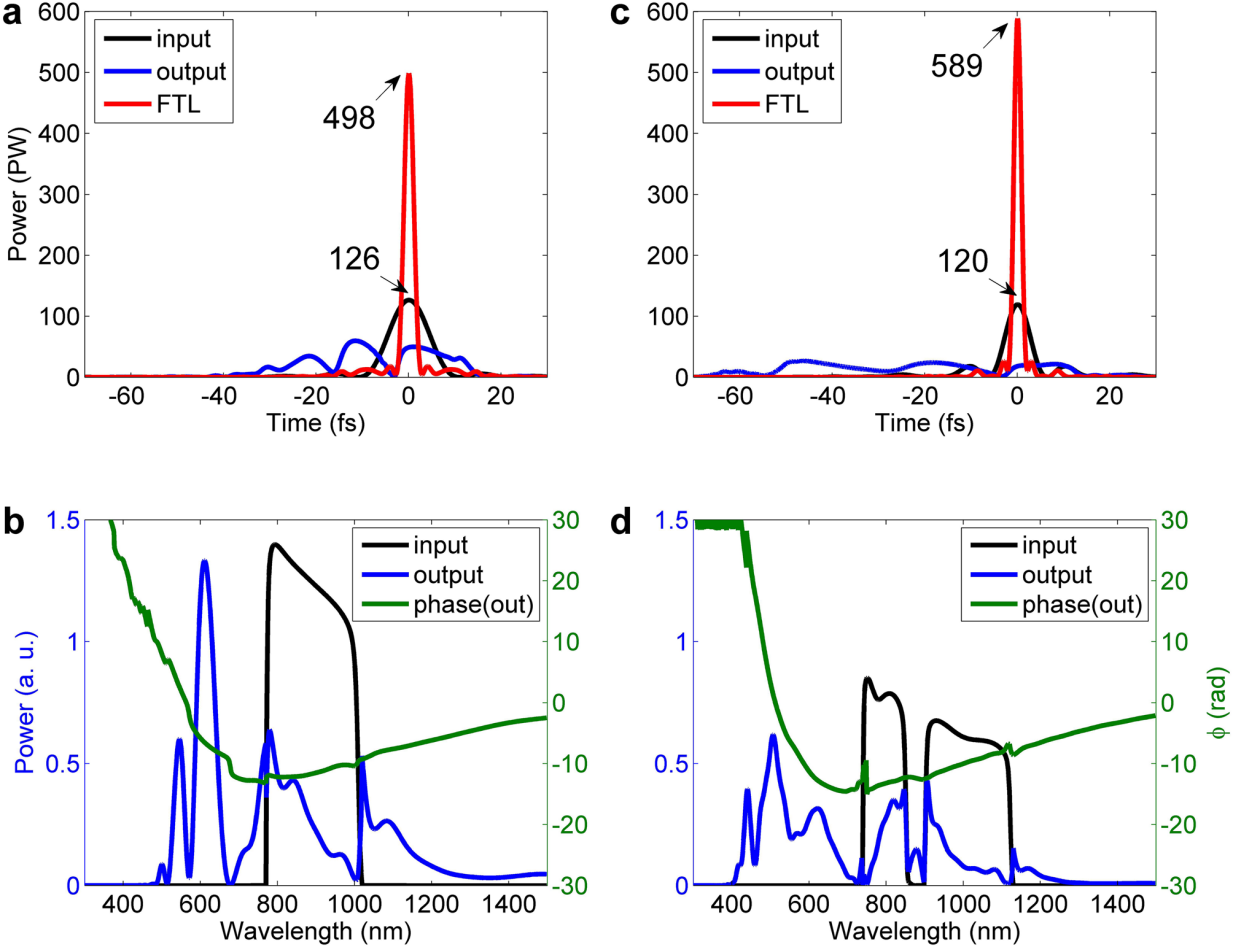
Input and output of thin wafer in time and spectrum. Post compression with a 0.5 mm-thick fused silica wafer for inputs from (**a**,**b**) OPCPA and (**c**,**d**) WNOPCPA. (**a**,**c**) show input (black), output (blue) and the FTL-compressed (red) pulses, and the unit of marked peak-powers is PW. **b**,**d**) show input (black) and output (blue) spectra and output spectral phase (green). FTL, Fourier-transform-limit, and PW, petawatt.

Figure 6 shows the pulses, the spectra, and the spectral phases after a 0.5 mm-thick fused silica for the input pulses from OPCPA (1358 J) and WNOPCPA (971 J), respectively. The FTL pulses which we can expect after the perfect dispersion compensation are also illustrated by red curves. In the OPCPA(1358 J)—post-compression, the expected peak-power of the FTL pulse will increase from 126 to 498 PW, because the pulse duration (FWHM) is reduced from around 11.0 fs to 2.7 fs (see Fig. 6a). And, in the WNOPCPA(971 J)—post-compression, the expected peak-power of the FTL pulse will increase from 120 to 589 PW, because the pulse duration (FWHM) is reduced from around 8.0 fs to ~ 1.65 fs (see Fig. 6c). This peak-power attained with the WNOPCPA—post-compression is higher than that of the OPCPA—post-compressions.

Comparison of Fig. 6b,d shows that the spectral broadening in the post-compression is significantly enhanced with WNOPCPA, resulting in a shorter pulse duration and then a higher peak-power of the compressed pulse, although the pulse energy of WNOPCPA is lower than that of OPCPA. The output spectral phases of the post-compression for two cases are illustrated, which have quite similar profiles.

### Dispersion compensation with chirped mirrors

The spectral phase (or dispersion) compensation by the chirped mirrors is a key process for this concept, since which directly determines the performance of the pulse compression and the peak-power. The spectral phase of a chirped pulse can be written as the Taylor expansion about the center angular frequency *ω*_0_ in the frequency domain,

1 $$\phi \left( \omega \right) = \phi_{0} + \phi_{1} \left( {\omega - \omega_{0} } \right) + \phi_{2} \left( {\omega - \omega_{0} } \right)^{2} + \phi_{3} \left( {\omega - \omega_{0} } \right)^{3} + \cdots ,$$

where, *ϕ*_0_, *ϕ*_*1*_, *ϕ*_*2*_ and *ϕ*_*3*_ are the derivatives of the phase with respect to the angular frequency *ω*, corresponding to the initial phase, the group delay, the group velocity dispersion (GVD) and the third order dispersion (TOD), respectively. The first two terms do not influence the pulse profile, whereas the second two of GVD and TOD do.

**Figure 7:**
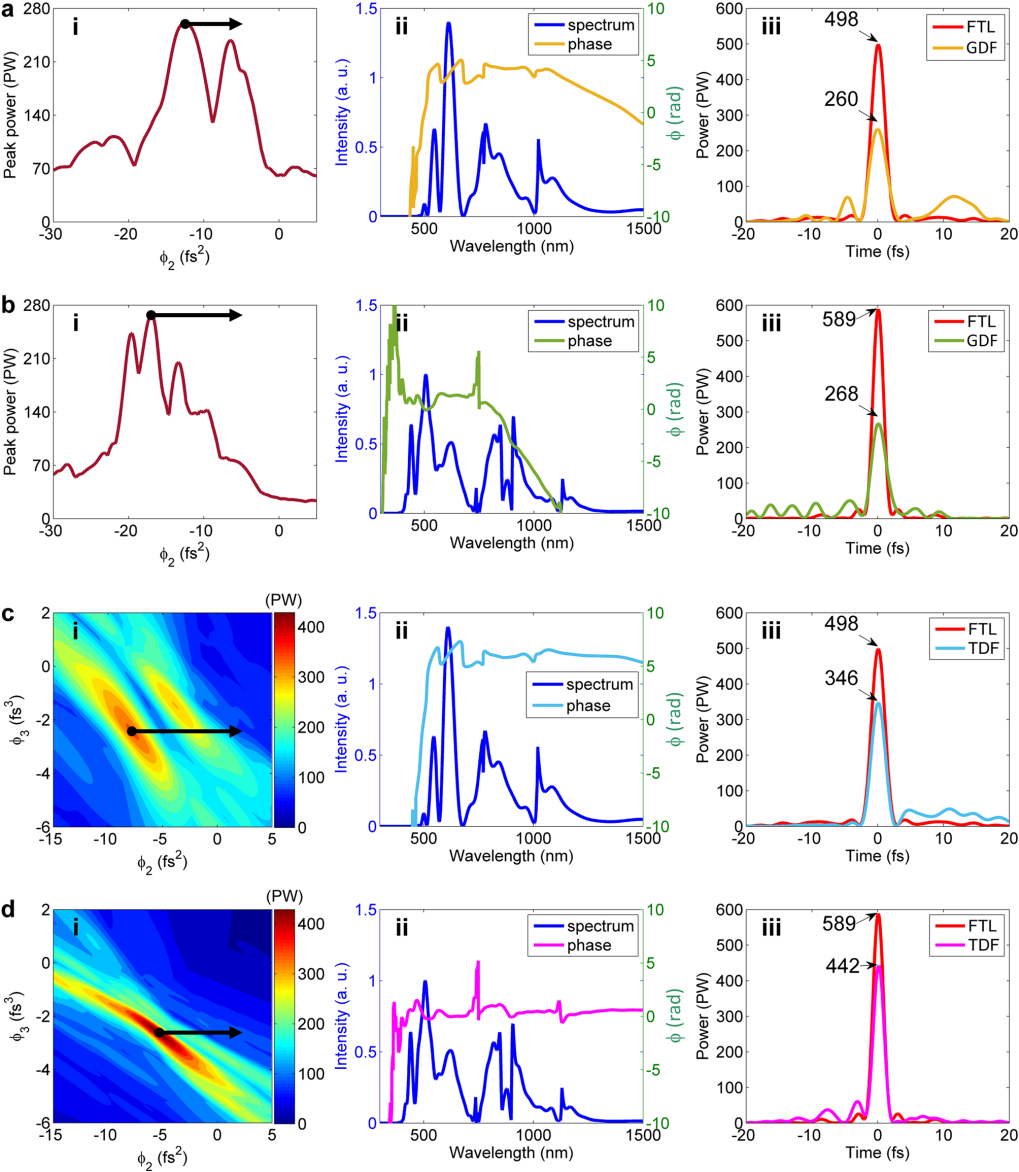
Different dispersion compensations with chirped mirrors. Dispersion compensation up to (**a**,**b**) GVD and (**c**,**d**) TOD for (**a**,**c**) OPCPA—post-compression and (**b**,**d**) WNOPCPA—post-compression, respectively. (i) shows dependence of peak-power on (**a**,**b**) GVD (*ϕ*_2_) and (**c**,**d**) GVD (*ϕ*_2_) and TOD (*ϕ*_3_), and the dispersion value for the highest peak-power is marked. (ii) shows spectrum (blue) and residual spectral phase of compressed pulse with the highest peak-power. (iii) shows compressed pulse corresponding to the highest peak-power, the FTL (red) pulse is given for reference, and the unit of marked peak-powers is PW. GVD, group velocity dispersion; TOD, third-order dispersion; FTL, Fourier-transform-limit; and PW, Petawatt.

In general, the chirped mirrors are used for broadband GVD control^31,59,60^. Using the spectra and spectral phases of the pulses after propagation in a 0.5 mm-thick fused silica shown in Fig. 6b for OPCPA (1358 J) and in Fig. 6d for WNOPCPA (971 J), we have evaluated the peak-powers of the compressed pulses by varying GVD, with the results shown in Fig. 7a(i),b(i), respectively. The pulse reaches the highest peak-power of 260 and 268 PW for the OPCPA(1358 J)—post-compression and WNOPCPA(971 J)—post-compression, respectively, when GVD is *ϕ*_*2*_ = -12.5 fs^2^ for OPCPA and *ϕ*_*2*_ = -17.0 fs^2^ for WNOPCPA, respectively. Figures 7(a)(ii) and 7(b)(ii) show the residual spectral phases as well as the spectra. Figure 7a(iii),b(iii) show the compressed pulses (here named as GVD-free (GDF) pulses) and the FTL pulses we can expect by the perfect dispersion compensation.

Recently the dispersion compensation up to TOD by chirped mirrors became possible since the TOD chirped mirrors can introduce net TOD without introducing any additional GVD^61^. Then, we have re-evaluated the peak-powers of the compressed pulses by varying both GVD and TOD for OPCPA (1,358 J) and for WNOPCPA (971 J), with the results shown in Fig. 7c(i),d(i), respectively. With the optimized highest peak-powers, Fig. 7c(ii), (iii), d(ii), (iii) show the spectra, residual spectral phases, compressed pulses [here named as TOD-free (TDF) pulses], and the FTL pulses we can expect by the perfect dispersion compensation. The TDF pulses reach their highest peak-powers of 346 and 442 PW for the OPCPA(1358 J)—post-compression and WNOPCPA(971 J)—post-compression at the combinations of (*ϕ*_*2*_ = − 7.7 fs^2^, *ϕ*_*3*_ = − 2.47 fs^3^ for OPCPA) and (*ϕ*_*2*_ = − 5.27 fs^2^, *ϕ*_*3*_ = − 2.64 fs^3^ for WNOPCPA), respectively.

In both cases of the dispersion compensation up to GVD or TOD, the WNOPCPA—post-compression results in a slightly higher peak-power, although WNOPCPA supplies a lower input pulse energy of 971 J than OPCPA does (1358 J). The result shows the clear advantage of a broader spectrum. However, the above highest peak-power of the optimized compressed pulses is still much lower than that of the FTL pulse. The FTL compressed pulse in technology right now could be generated by using the double (or more) chirped mirrors which can introduce well-controlled ultra-broadband dispersion to perfectly match a dispersion-distorted ultra-short pulse as introduced in Ref.^62^. In this case, the perfect pulse compressions illustrated by red curves in Fig. 7 are possible by implementing careful engineering.

## Discussion

### Limitation at the grating compressor

The grating compressor is the bottleneck restricting the development of the current PW lasers and the future EW lasers, where the low damage threshold of the gratings limits the energy fluence of laser beams. In this case, high-energy pulse compression requires very large gratings, affecting both optical engineering and economic cost. The damage mechanism shows the damage thresholds of current gratings for 8–15 fs ultrafast laser pulses, which center at the same wavelengths, are the same. Thereby, for the same compressed pulse energy limited by the grating size, a shorter pulse duration means a higher peak-power. This is the very reason why we choose the LBO crystal instead of the DKDP crystal here, because LBO can support a broader gain spectrum than DKDP, although DKDP (~ 400 mm) currently is larger than LBO (~ 150 mm). Here we are not challenging the approach of developing DKDP-based high-energy ultra-intense lasers, where the coherent beam combination is now tried to break through the limitation of the grating compressor.

### Damage and optical nonlinearity in chirped mirrors

For an EW-class laser, the damage and the optical nonlinearity in the chirped mirrors should be carefully considered. The potential damage of the chirped mirrors can be avoided by expanding the beam aperture, thus reducing the energy fluence. Since the beam aperture in the post-compression is 980 mm × 980 mm and 1160 mm × 1160 mm for the 971 J (WNOPCPA) and 1358 J (OPCPA) input, respectively, the energy fluence at the chirped mirrors is around 0.1 J/cm^2^ which is lower than the measured 1-on-1 damage threshold of 0.13 ± 0.01 to 0.28 ± 0.02 J/cm^2^^63^.

**Figure 8:**
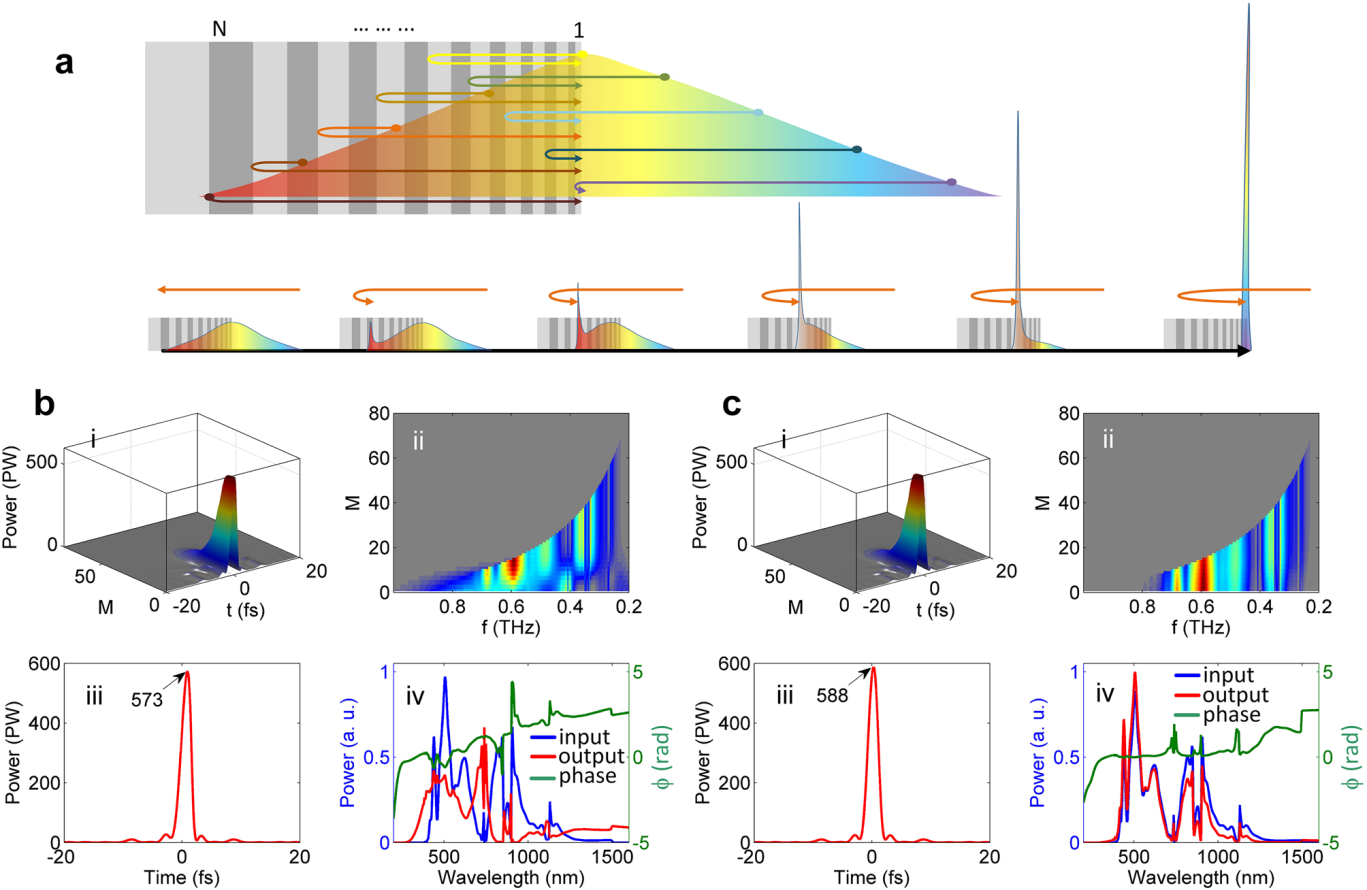
Nonlinearity in chirped mirror, and comparison. (**a**) Schematic diagram of pulse compression with a chirped mirror, where light with different wavelengths have different propagation paths. (**b,c**) show simulation results for WNOPCPA—post-compression with chirped mirrors composed of SiO_2_/TiO_2_ and SiO_2_/HfO_2_ layer pairs, respectively. In (**b,c**), (i) and (ii) illustrate evolutions of temporal and spectrum profiles of backward-propagating pulse in layers from *M* = 80 to 1, respectively, (iii) shows output pulse (the unit of marked peak-powers is PW), and (iv) shows input (blue) and output (red) spectra and output spectral phase (green). The layers are numbered as *M* = 1 to *M* = 80 from the surface to the deepest. PW, petawatt.

The optical nonlinear effect in a chirped mirror under irradiation of an ultra-intense, ultra-broadband laser pulse has not been well studied so far. Figure 8a schematically shows how a laser pulse composed of different wavelengths propagates in a chirped mirror, where different wavelength components propagate over different optical paths, thus experiencing different group delays [i.e., group delay dispersion (GDD)]. In this propagation, the optical nonlinearity in the chirped mirror structure could induce an extra spectral phase and also distort the spectral amplitude, thus affecting the pulse compression. The effect of the optical nonlinearity in a chirped mirror is estimated with the following simplified model. We assume that the chirped-mirror structure introduces the perfect GDD profile to match the dispersion of the input pulse. Figure 8a shows that the dispersion compensation begins with the reflection of the red component (the temporal leading edge) at the deepest layer. The reflected backward-propagating red component temporally and spatially overlaps with the shorter wavelength component in another layer. During the backward propagation, the bandwidth of the reflected pulse increases gradually and the dispersion of the input pulse is completely removed at the surface of the chirped mirror, where all wavelengths of the input pulse are contained in the reflected pulse. In the simulation, the material dispersion in the backward propagation of the reflected pulse is not included for simplification because the thickness of the chirped mirror is very small (generally 10–20 μm), and only GDD induced by the chirped Bragg structure is considered. Then we calculate the optical nonlinearity induced only by the reflected backward-propagating pulse, which has a sharp profile with very high peak-intensity, especially near the surface of the chirped mirror, as shown in Fig. 8a,b(i),c(i). The simulation result shown in Fig. 8b is based on an ideal chirped mirror of 80 alternating layers (i.e., 40 layer pairs) of SiO_2_ and TiO_2_, covering the wavelength region of 300–1500 nm. The SiO_2_ and TiO_2_ have the refractive indices of *n* = 1.4511 and 2.4920, and the nonlinear refractive indices of *n*_2_ = 3.67 × 10^–16^ and 9.4 × 10^–15^ cm^2^/W, respectively. The thickness of the layer pair is assumed to increase linearly from the surface layer pair to the deepest layer pair for simplification. Figure 8b(i) shows that the peak-power of the backward-propagating pulse increases slowly in the deep layers but increases rapidly as it propagates closer to the surface. Figure 8b(ii) shows that the bandwidth of the backward-propagating pulse gradually increases from the deepest layer to the surface layer, finally covering the whole spectrum of the input pulse. There are rapid spectral variations near surface from the *M* ~ 20 to *M* = 1 layers. Figure 8b(iii) shows the output pulse, and Fig. 8b(iv) shows the spectra of the input and output pulses and the output spectral phase. The peak-power is reduced from 589 PW to around 573 PW due to distortions of both spectral amplitude and phase (arising from SPM mainly in the TiO_2_ layers), especially the spectral phase at the longer wavelength region which experiences longer propagation.

This adverse effect can be reduced by replacing TiO_2_ by HfO_2_, which has *n* = 2.0851 and *n*_2_ = 5.8 × 10^–16^ cm^2^/W, respectively. Note that *n*_2_ of HfO_2_ is about 16 times smaller than that of TiO_2_. The simulation results are shown in Fig. 8c. Comparing Fig. 8c(i), (ii) with Fig. 8b(i), (ii) respectively, the peak-power of the backward-propagating pulse reaches a higher value, and the spectral variation near the surface (e.g., from *M* ~ 20 to *M* = 1 layers) disappears. Figure 8c(iii) shows that the compressed pulse almost has no distortion, reaching the nearly ideal peak-power of 588 PW. Figure 8c(iv) shows that the difference between the input and the output spectra is negligible and the spectral phase distortion is small. Based on these comparisons, we conclude that low-nonlinearity-material (e.g., SiO_2_/HfO_2_) chirped mirrors are more suitable for the ultra-intense pulse compression.

### Spatial intensity modulation in post-compression

In the above, it is assumed that the input laser to the thin-plate post-compression has a stable pulse energy and a flat-top beam. Here, we consider the effects of the pulse energy fluctuation and the spatial intensity non-uniformity on the post-compression.

**Figure 9:**
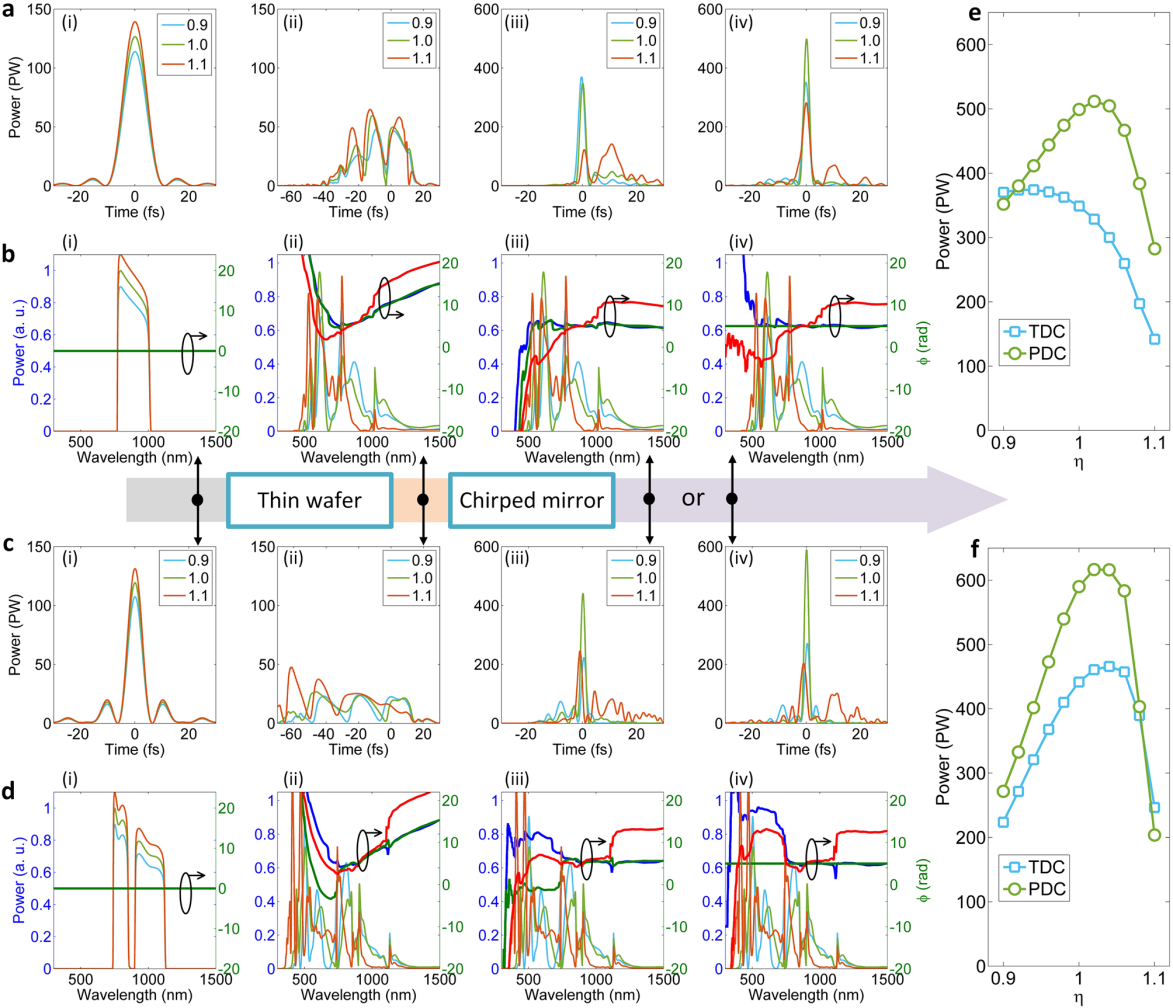
Pulse energy fluctuation effect. For (**a**,**b**) OPCPA—post-compression and (**c**,**d**) WNOPCPA—post-compression, when relative intensity changes induced by input energy fluctuation are *η* = 0.9, 1 and 1.1, (**a**,**c**) pulse and (**b**,**d**) spectrum and phase at different propagation positions, (i) at input, (ii) after thin wafer, (iii) after chirped mirrors [with TOD dispersion compensation (TDC) at *η* = 1], and (iv) after chirped mirrors [with perfect dispersion compensation (PDC) at *η* = 1]. Peak-power of TDC and PDC compressed pulses as a function of *η* for (**e**) OPCPA—post-compression and (**f**) WNOPCPA—post-compression, respectively.

We first consider the pulse energy fluctuation, and show the simulation results for the OPCPA-post-compression and the WNOPCPA—post-compression in Fig. 9a–d, respectively, for comparison. When the input laser has ± 10% energy fluctuation, the relative input intensity changes as *η* = 0.9, 1 and 1.1, where *η* = 1 corresponds to the ideal case [i.e., Figs. 6(a), 6(b) and 6(c), 6(d) for two configurations, respectively]. Figure 9a,c show pulses, and Fig. 9b,d show the corresponding spectra and spectral phases. Subfigures (i), (ii), (iii), and (iv) in Fig. 9a–d respectively show the results at the input, after nonlinear propagation in the 0.5 mm-thick fused silica thin wafer, after the chirped mirror with up to TOD dispersion compensation at *η* = 1, and after the chirped mirror with perfect dispersion compensation at *η* = 1, where the nonlinear propagation is changed by the change of the input intensity. Here, we assume chirped mirrors introduce spatially uniform dispersion compensation at *η* = 1. Subfigures (iii) and (iv) in Fig. 9a,c show that the peak-power is reduced, no matter when *η* is increased to 1.1 or reduced to 0.9. This mainly arises from the change of the spectral phase and the spectrum due to the change of the input intensity [see Fig. 9b(ii),d(ii)], which breaks the dispersion-matching with the chirped mirrors [see (iii) and (iv) in Fig. 9b,d]. Figure 9e,f show the dependence of the final peak-power (with both TOD and perfect dispersion compensations at *η* = 1) on the relative input intensity change *η* induced by the input energy fluctuation for two configurations. Comparing with the OPCPA-post-compression, although the sensitivity is higher for the WNOPCPA—post-compression, the averaged peak-power for different shots linearly varying from *η* = 0.9 to 1.1 is still stronger. With TOD dispersion compensation at *η* = 1, the averaged peak-power is 312 PW for the OPCPA-post-compression and 428 PW for the WNOPCPA-post-compression; and with perfect dispersion compensation at *η* = 1, which is 369 PW for the OPCPA-post-compression and 458 PW for the WNOPCPA-post-compression. If the energy fluctuation can be well controlled, e.g., ± 5%, the performance of the WNOPCPA—post-compression can be even better.

**Figure 10:**
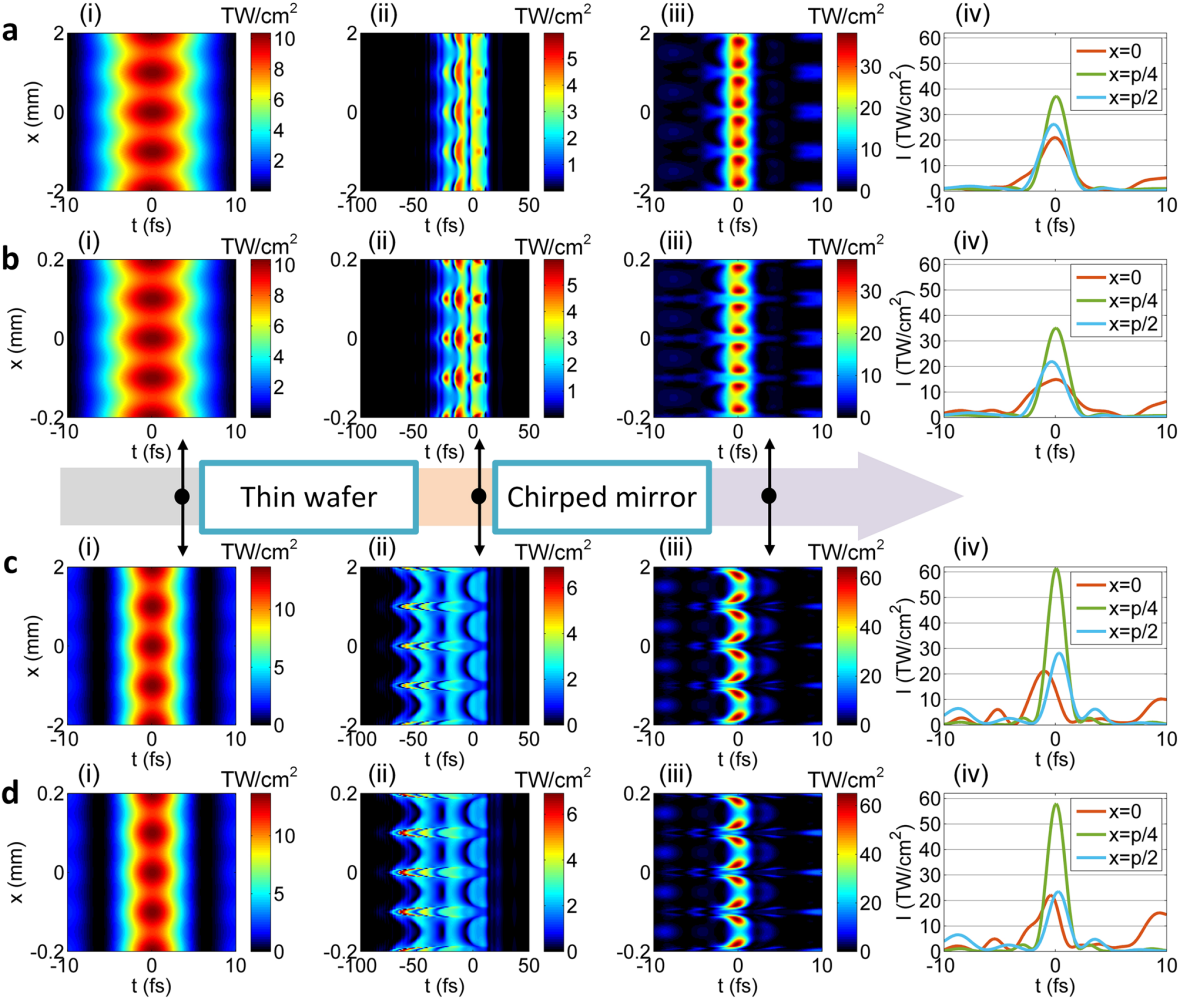
Spatial intensity non-uniformity effect. For (**a**,**b**) OPCPA—post-compression and (**c**,**d**) WNOPCPA—post-compression, when input beam has (**a**,**c**) slow- or (**b**,**d**) fast-variation spatial intensity non-uniformity, spatiotemporal intensity distributions at different propagation positions (i) at input, (ii) after thin wafer, and (iii) after chirped mirror (with perfect dispersion compensation at *η* = 1). Spatial intensity non-uniformity has a cosine-function-like profile varies between 0.9 to 1.1 (i.e., ± 10%) in space, *p* is the spatial period, which equals (**a**,**c**) 1 mm and (**b**,**d**) 0.1 mm, respectively. (iv) shows compressed pulses at three positions of *x* = 0, *p*/4, and *p*/2 across beam, corresponding to relative intensity changes of *η* = 1.1, 1 and 0.9, respectively.

We next consider the spatial intensity non-uniformity, and the simulation results for the OPCPA—post-compression and the WNOPCPA—post-compression are shown in Fig. 10a,b and Fig. 10c,d, respectively, for comparison. The modulation of the spatial intensity non-uniformity is assumed to have a cosine-function-like profile of *M*⋅cos(*x*/*p*) + 1, where *M* is a coefficient, *x* is the transverse coordinate, and *p* is the spatial period. When the coefficient *M* is 0.1, the relative intensity change *η* across the beam aperture varies between 0.9 and 1.1 (i.e., ± 10%), where *η* = 1 corresponds to the ideal case (i.e., Fig. 6a–d for two configurations, respectively). The spatial period *p* in Fig. 10a–d is 1 mm (i.e., slow-spatial variation) and 0.1 mm (i.e., fast-spatial variation), respectively, showing small-scaled self-focusing in different spatial scales. Subfigures (i), (ii), and (iii) in Fig. 10a–d respectively show the 2-D spatiotemporal intensity distributions at the input, after the nonlinear propagation in the 0.5 mm-thick thin fused silica wafer, and after the chirped mirror with spatially uniform perfect dispersion compensation at *η* = 1. Subfigures (iv) in Fig. 10a–d show three compressed pulses at three typical positions across the beam aperture. Subfigures (i) and (ii) in Fig. 10a–d show the spatial intensity non-uniformity after the fused silica is increased, which arises from different nonlinear propagations induced by different input intensities. Subfigures (ii) and (iii) in Fig. 10a–d show the spatial intensity non-uniformity after the chirped mirror is further increased, which arises from dispersion-mismatching with the chirped mirror. Subfigures (iv) in Fig. 10a–d show, at the positons of *x* = 0, *p*/4, and *p*/2 (i.e., the relative intensity changes of *η* = 1.1, 1.0, and 0.9), whether the increasing or the decreasing of the intensity distorts the compressed pulse. Because the change of the intensity changes nonlinear propagation in the fused silica, which breaks dispersion-matching with the following chirped mirror. Comparing Fig. 10 (c)(iv) with Fig. 10(a)(iv) or Fig. 10(d)(iv) with Fig. 10(b)(iv), the distorted pulses for two configurations have almost the same peak-values, although the sensitivity is higher for the WNOPCPA—post-compression. By integrating the peak-intensity across the beam aperture, for the slow-spatial variation of *p* = 1 mm, the average power-power is 403 PW for the OPCPA-post-compression and 420 PW for the WNOPCPA-post-compression; and for the fast-spatial variation of *p* = 0.1 mm, which is 355 PW for the OPCPA-post-compression and 389 PW for the WNOPCPA-post-compression. The WNOPCPA-post-compression still has a slightly better performance. The difference between Fig. 10a,b or Fig. 10c,d, i.e., between slow-spatial variation (*p* = 1 mm) and fast-spatial variation (*p* = 0.1 mm), is due to small-scaled self-focusing, which further distorts the compressed pulses [comparing Fig. 10b with Fig. 10a or Fig. 10d with Fig. 10c. The spatial frequency of small-scaled self-focusing that can generate obvious degradation is very high (e.g., *p* = 0.1 mm here), which can be reduced by adding a spatial filter before the post-compression (see details in Ref.^31^).

Finally, it is noted that the spectral phase of nonlinear propagation in the thin fused silica wafer is dynamical (influenced by the input intensity), while that of the chirped mirrors is constant, and the result is the dispersion-matching between nonlinear propagation and chirped mirror is broken. This shows that the pulse energy fluctuation and the spatial intensity non-uniformity of the input laser beam have to be highly controlled. Here, the pulse compression is much more sensitive to the change in the spectral phase than the spectral amplitude, although the intensity change also influences the output spectrum.

### Other engineering considerations

In the WNOPCPA presented in this paper, we have reduced the number of pump-beamlets from four in our previous work^45^ to only two. This leads to two advantages: the pump interference is avoided completely and the conversion efficiency from the pump to the signal is improved. The amplified pulse with two separated spectra enhances the spectral broadening in the post-compression, eventually generating a continuous ultra-broadband spectrum. We note that the coherence between the two separated amplified-spectra is maintained since they directly come from a single coherent pulse and no extra spatial or temporal separation (e.g., beam or pulse separation and/or combination) is introduced.

As discussed in the above, the precise dispersion compensation by chirped mirrors for generation of the FTL pulse in the post-compression is essential and feasible. The fabrication of the meter-sized chirped mirrors is still an engineering challenge, although the combination of double (or more) chirped mirrors could realize the required perfect dispersion compensation.

Also, as discussed in the above, the pulse energy fluctuation and the spatial intensity non-uniformity of the laser beam have to be well controlled to reduce the nonlinear phase variation induced in the post-compression. In order to achieve a flattop signal beam in OPCPA and/or WNOPCPA, the beam smoothing technologies should be considered in the pump beam in the future^64^.

There are other potential problems to realize a sub-optical-cycle EW-class laser. In additions to the previously well-studied issues such as dispersion-control, wave-front-control, high-gain/energy-amplification, spectral-narrowing, temporal-contrast, beam-smoothing, beam-pointing, beam-focusing, etc., more recent issue of spatiotemporal/spectral coupling distortion induced by deformation of the compressor gratings^65–68^ should be addressed, although it is technically controllable^69^.

## Conclusion and outlook

We have proposed and simulated an ultra-broadband concept for an EW-class peak-power by combining the (two-beamlet pumped) WNOPCPA with the thin-plate post-compression. Detailed numerical analyses of the proposed 0.5 EW laser concept, which is composed of the state-of-the-art optical components, have been presented including optical parametric amplification in the five-stage amplifiers, evolution of the spectral broadening and the spectral phase in a thin fused silica wafer, and dispersion compensation by the chirped mirrors. Effects of the nonlinearity in the chirped mirror and the spatial intensity modulation in the post-compression are discussed. Compared with the OPCPA—post-compression configuration, a much broader spectrum and accordingly an even shorter pulse is generated by the WNOPCPA—post-compression configuration. This concept will be capable of generating an ultra-short, high-energy, and ultrahigh peak-power laser pulse with the spectral coverage of 0.4–1.3 μm, the pulse duration (FWHM) of only ~ 1.65 fs, the pulse energy of 971 J, and accordingly the peak-power of 589 PW.

The conceptual design of the 0.5 EW laser proposed in this paper is based on the largest available size of—the type-I LBO crystal (130 mm × 130 mm) and the surface damage threshold of the LBO crystal (~ 20 GW/cm^2^) which limits the pump energy in the final amplifier. In future, a real EW laser could be achieved when larger LBO crystals, gratings, and other optics become available and/or by coherent addition of the two 0.5 EW beams described in this paper. An EW-class laser will be a very complex system, and a lot of engineering problems need to be considered in the future. Fortunately, since the sub-optical-cycle (less than 3 fs) intense laser pulses have been successfully demonstrated in small-scale laser systems worldwide (see Table 1), the next step of dramatically scaling the peak-power/intensity to an extremely high level is theoretically feasible.

## Methods

### Simulation of WNOPCPA

Refer to the theoretical model of the two-beam-pumped non-collinear OPCPA^44^, the interaction of one signal light, two pump lights and two idler lights satisfy the five-wave coupled-wave equations, and, in the frequency domain, which is given by^45^

2 $$\begin{gathered} \frac{{\partial A_{s} }}{\partial z} + ik_{s} A_{s} = - i\sum\limits_{m = 1}^{2} {\frac{{\chi_{m}^{(2)} \omega_{s} }}{{n_{s} c}}A_{p,m} A_{i,m}^{*} } , \hfill \\ \frac{{\partial A_{p,1} }}{\partial z} + ik_{p,1} \cos \alpha_{1} A_{p,1} = - i\frac{{\chi_{1}^{(2)} \omega_{p} }}{{n_{p,1} c\cos \alpha_{1} }}\frac{1}{{\cos^{2} \left( {\alpha_{1} - \rho } \right)}}A_{s} A_{i,1} , \hfill \\ \frac{{\partial A_{i,1} }}{\partial z} + ik_{i,1} \cos \left( {\alpha_{1} + \beta_{1} } \right)A_{i,1} = - i\frac{{\chi_{1}^{(2)} \omega_{i,1} }}{{n_{i,1} c\cos \left( {\alpha_{1} + \beta_{1} } \right)}}A_{p,1} A_{s}^{*} , \hfill \\ \frac{{\partial A_{p,2} }}{\partial z} + ik_{p,2} \cos \alpha_{2} A_{p,2} = - i\frac{{\chi_{2}^{(2)} \omega_{p} }}{{n_{p,2} c\cos \alpha_{2} }}\frac{1}{{\cos^{2} \left( {\alpha_{2} - \rho } \right)}}A_{s} A_{i,2} , \hfill \\ \frac{{\partial A_{i,2} }}{\partial z} + ik_{i,2} \cos \left( {\alpha_{2} + \beta_{2} } \right)A_{i,2} = - i\frac{{\chi_{2}^{(2)} \omega_{i} }}{{n_{i} c\cos \left( {\alpha_{2} + \beta_{2} } \right)}}A_{p,2} A_{s}^{*} , \hfill \\ \end{gathered}$$

where, *k*_s_ *k*_p,m_ and *k*_i,m_ are the wave vectors of signal, number m pump and number m idler (i.e., full dispersions of the material are considered), *χ*_m_ is the number m second-order nonlinear coefficient which is direction-dependent, and *n* is the refractive index which is wavelength and direction dependent, *α*_m_ is the number m non-collinear angle between signal and number m pump (i.e., non-collinear angle is considered), *β*_m_ is the number m angle between number m pump and number m idler which is also wavelength dependent, *ρ* is the pump walk-off angle (i.e., pump walk-off is considered).

### Simulation of post-compression

In the spatiotemporal domain, the propagation of an ultra-short pulse in a fused silica thin wafer can be approximately described by^70^

3 $$\frac{\partial A}{{\partial z}} = - \left( {i\frac{{\beta_{2} }}{2}\frac{{\partial^{2} }}{{\partial t^{2} }} - \frac{{\beta_{3} }}{6}\frac{{\partial^{3} }}{{\partial t^{3} }} + \frac{\alpha }{2}} \right)A + \frac{i}{{2n_{0} k_{0} }}\left( {\frac{{\partial^{2} }}{{\partial x^{2} }} + \frac{{\partial^{2} }}{{\partial y^{2} }}} \right)A + i\gamma \left( {\left| A \right|^{2} + \frac{i}{{\omega_{0} A}}\frac{{\partial \left( {\left| A \right|^{2} A} \right)}}{\partial t} - T_{R} \frac{{\partial \left| A \right|^{2} }}{\partial t}} \right)A,$$

where *z* is the propagation coordinate, *x* and *y* are the transverse coordinates, *A*(*t*, *z*) is the slowly varying amplitude of the electric field at time *t* and position *z*, *β*_2_ is GVD, *β*_3_ is TOD, *α* is the attenuation coefficient of the material, *γ* = 2*n*_*0*_*ε*_*0*_*ck*_*0*_*n*_*2*_ is the strength of the material nonlinearity (with *n*_*0*_ refractive index, *ε*_*0*_ vacuum permittivity, *k*_*0*_ wavenumber, and *n*_*2*_ the nonlinear refractive index), *ω*_0_ is the center angular frequency, and *T*_*R*_ is the Raman response time. The effects of dispersion, attenuation, diffraction, self-focusing, SPM, self-steepening and SRS are considered. When considering the pulse energy fluctuation or the spatial intensity non-uniformity, a coefficient of *η* or *η*(*x*, *y*) is added in the initial input intensity of *I*(*x*, *y*, *z* = 0, *t*), respectively.

## Supplementary Information

Supplementary Information.

## Data Availability

The data that support the findings of this study are available from the corresponding author upon reasonable request.
