# Supplementary material for: Simulating an ultra-broadband concept for Exawatt-class lasers
Source: Sci Rep. 2021 Jan 8;11:151. doi: 10.1038/s41598-020-80435-6 (PMC7794522; doi:10.1038/s41598-020-80435-6)
Supplement: Supplementary file 1 — Supplementary Information. [file 41598_2020_80435_MOESM1_ESM.docx]

**Supplement:**

**Simulating an ultra-broadband concept for Exawatt-class lasers**

Zhaoyang Li,* Yoshiaki Kato, and Junji Kawanaka

Institute of Laser Engineering, Osaka University, 2-6 Yamadaoka, Suita, Osaka 565-0871, Japan

*zhaoyang-li@ile.osaka-u.ac.jp

**Phase-matching optimization for OPCPA and WNOPCPA**. The phase-matchings of optical parametric chirped-pulse amplification (OPCPA) and wide-angle non-collinear optical parametric chirped-pulse amplification (WNOPCPA) are optimized for ultra-broadband gain spectra based on the 527 nm pumped type-I LBO crystal. The *x*-*y* plane is chosen as the phase-matching plane (*θ* = 90°), and Fig. 1(a) shows the phase-matching curves for different pump angles of *φ*_p_ = 12.90°, 13.12° and 14.50°. When *φ*_p_ = 14.50°, Fig. 1(a) shows a plat curve is obtained, corresponding to the broadest gain spectrum, which is chosen as the phase-matching for OPCPA (*φ*_p_ = 14.50°, *φ*_s_ = 13.10°). The black line in Fig. 1(b) shows the gain spectrum using the analytical formula when the pump intensity is 2 GW/cm^2^ and the crystal length is 10 mm, and the black line in Fig. 1(c) show the gain spectrum using the coupled wave-equation in the main article when saturation effect is considered. Figure 1(a) shows the phase-matching of *φ*_p_ = 12.90° & 13.12° and *φ*_s_ = 12.00° is optimized for WNOPCPA, and two broadband spectral components corresponding to two pump beamlets enhance the overall gain spectrum. The colored lines in Figs. 1(b) and 1(c) show the gain spectra using the analytical formula (with the pump intensity 2 GW/cm^2^ and the crystal length 10 mm) and using the coupled wave-equation (with the saturation effect and the pump-signal temporal overlap) in the main article.


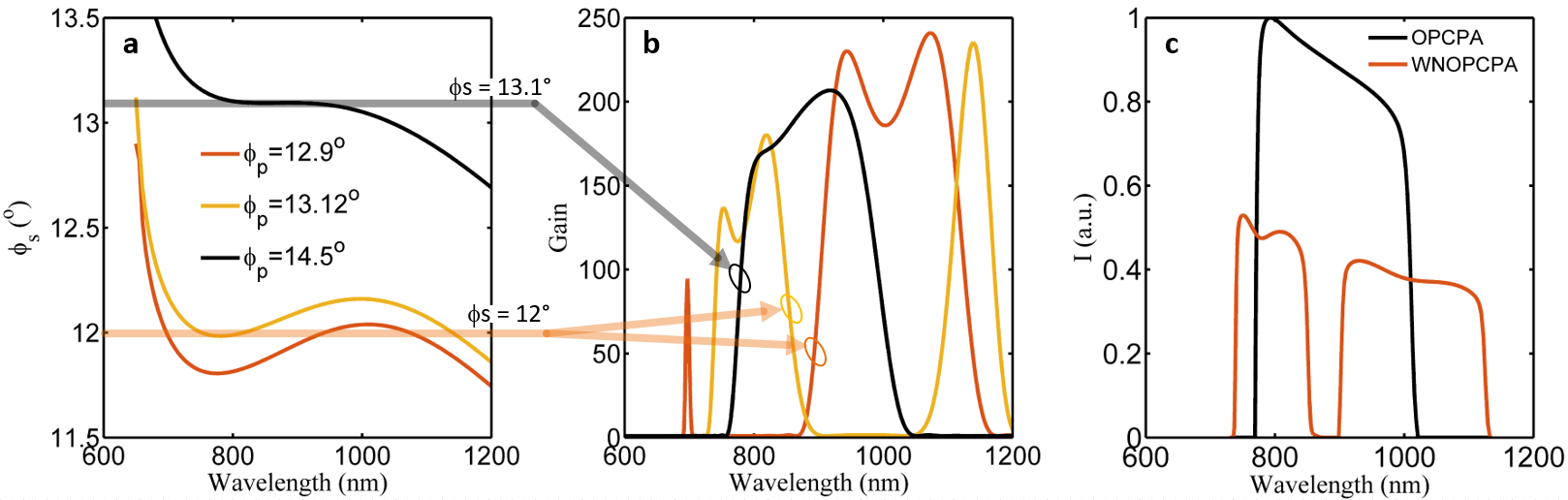


**Figure 1.** **Phase-matching optimization, and gain spectra of OPCPA and WNOPCPA**. (**a**) Phase-matching curves for different pump angles of *φ*_p_ = 12.90°, 13.12° and 14.50° in *x-y* plane of 527 nm pumped type-I LBO crystal. Gain spectra calculated using (**b**) analytical formula and (**c**) coupled wave-equation.

**Broadband amplification with OPCPA.** The three-wave (signal, pump and idler) optical parametric coupling is simulated using the following parameters. In the spectral domain, the pump wavelength is 527 nm and the spectrum of the signal covers at least 765-1020 nm. In the temporal domain, the pulse duration of both pump and signal is 1 ns. In the spatial domain, the signal and pump beams in Amp 1-Amp 5 have flattop square apertures with a side length of 5, 20, 130, 130 and 130 mm, respectively. The total energies of the pumps for Amp 1-Amp 5 are 0.3, 10, 500, 1000 and 2000 J, respectively. All amplifiers are based on the type-I LBO (biaxial) crystal, and the x-y plane is chosen as the phase-matching plane (*θ* = 90°). The propagation angles *φ* (angles from the optical x-axis) of the pump and signal beams are 14.50° and 13.10° respectively. These parameters together with other parameters are summarized in Table 1.

Table 1. Parameters in each amplifier from Amp 1 to Amp 5.

|  | Amp 1 | Amp 2 | Amp 3 | Amp 4 | Amp 5 |
| --- | --- | --- | --- | --- | --- |
| Crystal type | Type I LBO | | | | |
| Crystal aperture (mm^2^) | 5×5 | 20×20 | 130×130 | 130×130 | 130×130 |
| Crystal length (mm) | 17 | 8.5 | 8.5 | 3 | 2 |
| Pump spectrum (nm) | 527 | | | | |
| Pump angle (°) | *θ* = 90, *φ* = 14.5 | | | | |
| Pump duration (ns) | 1 | | | | |
| Pump energy (J) | 0.3 | 10 | 500 | 1000 | 2000 |
| Pump intensity (GW/cm^2^) | 1.2 | 2.5 | 2.96 | 5.92 | 11.83 |
| Signal spectrum range (nm) | 765-1020 | | | | |
| Signal angle (°) | *θ* = 90, *φ* = 13.1 | | | | |
| Signal duration (ns) | 1 | | | | |
| Input signal energy (J) | 0.0001 | 0.14 | 5.2 | 264 | 813 |
| Output signal energy (J) | 0.14 | 5.2 | 264 | 813 | 1,940 |


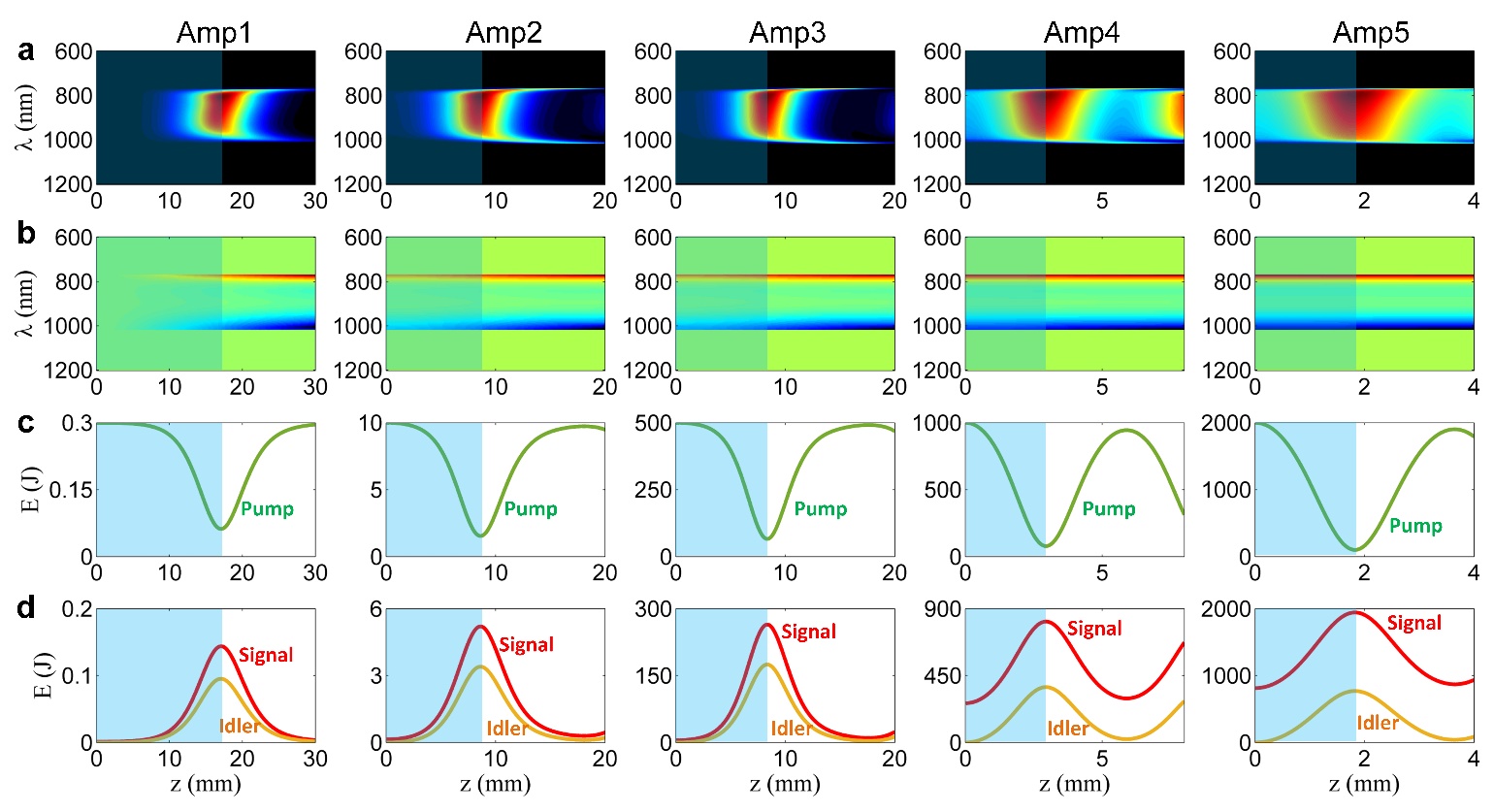


**Figure 2.** **Evolution of the OPCPA amplification chain.** Evolutions of (**a**) spectrum and (**b**) nonlinear spectral phase of signal, and energies of (**c**) pump and (**d**) signal and idler in nonlinear crystals along propagation *z* from Amp 1 to Amp 5. Blue areas show the optimized crystal lengths.

Figures 2(a)-2(d) show the simulation results of the evolutions of the spectral intensity and the nonlinear spectral phase of the signal beam, and the energies of the pump, signal and idler beams along propagation in the nonlinear crystals. The linear phases due to dispersion in the stretcher and the crystal material have been removed in Fig. 2(b), in order to directly observe the evolution of the nonlinear spectral phase of the signal. As shown in Fig. 2(d), the energy of the signal beam becomes maximum of 0.14, 5.2, 264, 813 and 1,940 J in Amp 1-Amp 5 at the crystal length of *z* = 17, 8.5, 8.5, 3 and 2 mm, respectively. The energy of the signal beam at the output of Amp 5 reaches 1,940 J, which is 55 % of the total pump energy of 3,510 J.


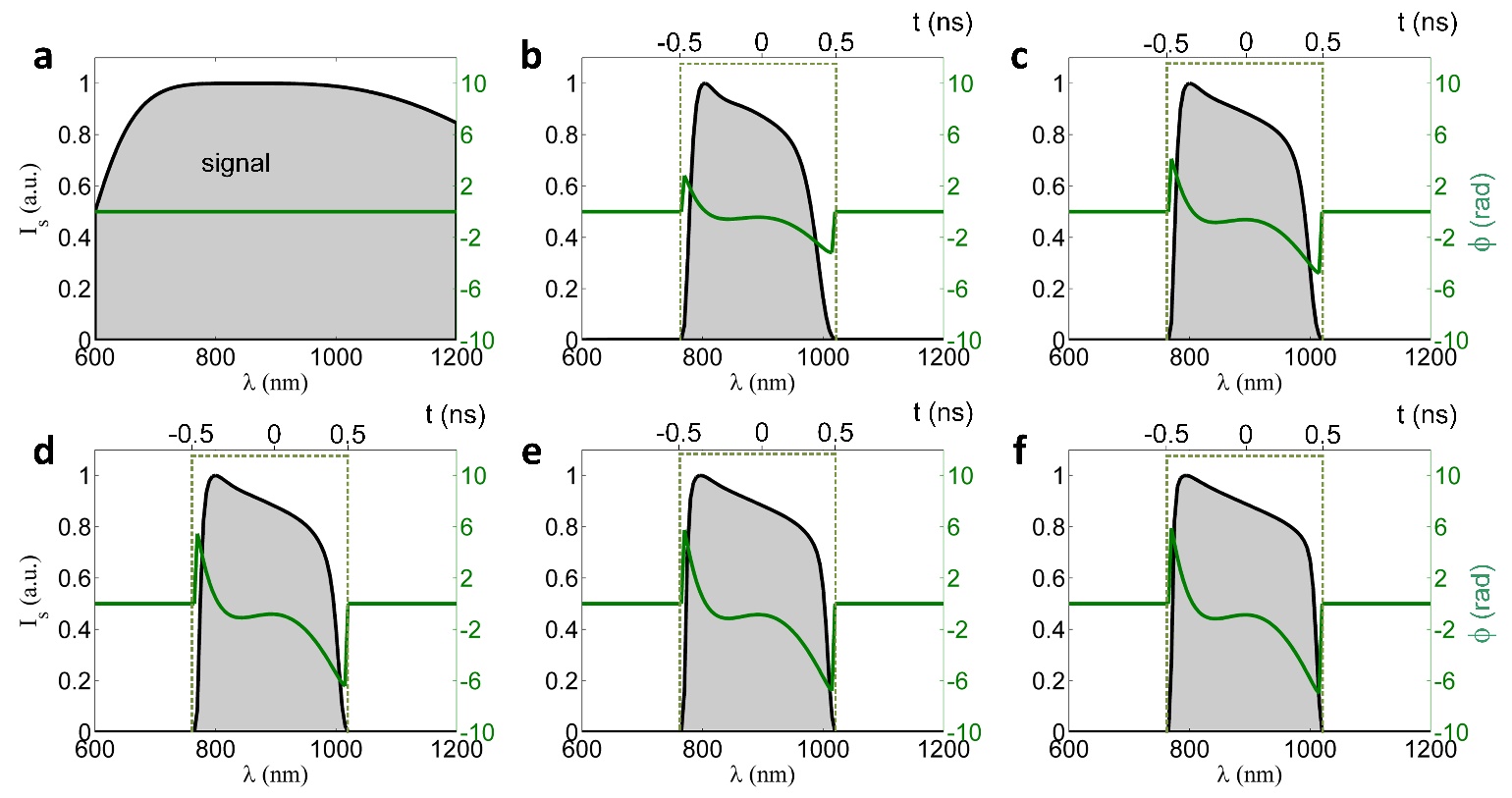


**Figure 3.** **Spectrum and nonlinear spectral phase at each stage.** Spectrum (black) and nonlinear spectral phase (green) of signal at (**a**) input and (**b**)-(**f**) outputs of Amp 1-5, respectively. Chirped signal pulses and two pump pulses in time are illustrated by upper labels.

Figure 3 shows the spectra and the nonlinear spectral phases of the signal beam at the input to Amp 1 (i.e., output of the front-end) and at the outputs of Amp 1-Amp 5. The amplified signal has a broadband spectrum of around 765-1020 nm. The nonlinear spectral phase increases gradually from Amp 1 to Amp 5 and becomes approximately 4π rad (in peak-to-valley) at the output, and this spectral phase distortion is within the control range of the available device for spectral phase control. In the pulse compression, most of the temporal dispersion and the residual high-order dispersion is removed by the nano-second grating compressor and the spectral phase controlling device at the front-end, respectively. When with the same 70% throughput efficiency nano-second grating compressor, a Fourier-transform-limit pulsed beam with a pulse energy of 1,358 J, a pulse duration (FWHM) of ~11.0 fs, and a beam aperture of 1160 mm × 1160 mm is injected to the 0.5 mm-thick fused silica post-compressor for comparison with the case of WNOPCPA in the main article.
